# Fecal Near Infrared Spectroscopy to Discriminate Physiological Status in Giant Pandas

**DOI:** 10.1371/journal.pone.0038908

**Published:** 2012-06-13

**Authors:** Erin E. Wiedower, Andrew J. Kouba, Carrie K. Vance, Rachel L. Hansen, Jerry W. Stuth, Douglas R. Tolleson

**Affiliations:** 1 Grazingland Animal Nutrition Lab, Blackland Research and Extension Center, Texas A&M University, Temple, Texas, United States of America; 2 Department of Conservation and Research, Memphis Zoological Society, Memphis, Tennessee, United States of America; 3 Department of Biochemistry and Molecular Biology, Mississippi State University, Mississippi, United States of America; 4 Department of Rangeland Ecology and Management, Texas A&M University, College Station, Texas, United States of America; 5 School of Natural Resources and the Environment, V Bar V Ranch, The University of Arizona, Camp Verde, Arizona, United States of America; Monash University, Australia

## Abstract

Giant panda (A*iluropoda melanoleuca*) monitoring and research often require accurate estimates of population size and density. However, obtaining these estimates has been challenging. Innovative technologies, such as fecal near infrared reflectance spectroscopy (FNIRS), may be used to differentiate between sex, age class, and reproductive status as has been shown for several other species. The objective of this study was to determine if FNIRS could be similarly used for giant panda physiological discriminations. Based on samples from captive animals in four U.S. zoos, FNIRS calibrations correctly identified 78% of samples from adult males, 81% from adult females, 85% from adults, 89% from juveniles, 75% from pregnant and 70% from non-pregnant females. However, diet had an impact on the success of the calibrations. When diet was controlled for plant part such that “leaf only” feces were evaluated, FNIRS calibrations correctly identified 93% of samples from adult males and 95% from adult females. These data show that FNIRS has the potential to differentiate between the sex, age class, and reproductive status in the giant panda and may be applicable for surveying wild populations.

## Introduction

Giant pandas (*Ailuropoda melanolueca*) are listed as critically endangered by the International Union for the Conservation of Nature (IUCN) [1] with an estimated 1,600 animals scattered across six mountain ranges and three provinces in central Asia [2]. There are also currently over 330 captive individuals kept in 50 breeding facilities and zoos worldwide [3]. Fragmented habitat and a growing number of anthropogenic influences threaten the remaining free-range populations and restrict interaction of wild pandas between protected areas.

Having a clear understanding of the panda population in the wild and demographics within individual nature reserves is critical for establishing management policies and appropriate levels of protection. However, there are numerous challenges to obtaining surveys for giant pandas in the wild. First, pandas occupy extremely mountainous areas with steep terrain such that conducting traditional habitat transects is very difficult. Second, the few pandas left are scattered across a large geographic area and are by nature extremely secretive [4]. Additionally, it is difficult to determine the number of unique individuals using non-invasive methods without also applying more costly molecular genetic analysis [5],[6]. Although observing pandas in the wild is difficult, locating panda sign (e.g. feces) is much easier owing to the 10 to18 kg of bamboo they eat each day [4] resulting in numerous fecal boli passed. Work by Schaller [4] and Pan et al. [7] suggest that the bite size of undigested bamboo leaves or culm in feces may be specific to individuals.

Over the last decade, significant progress has been made in developing genetic markers that have provided more current information on population estimates in specific nature reserves [8], [9],[10]. Genetic analysis has provided a wealth of information on gene flow and diversity within and between fragmented populations [8],[9] and can also be used to identify numbers and sex of individuals [5],[10]. While genetic analysis is a useful tool, the amount of effort and expense for processing samples is quite high. However, there may be other technologies that can help support wild panda population surveys. For example, near infrared reflectance spectroscopy (NIRS) could be applicable to sex and age determination, and perhaps even pregnancy status or individual identification.

Near infrared spectroscopy is a non-invasive analytical technique that has been widely used in agriculture to predict forage characteristics of domestic [11], as well as wild herbivores [12],[13],[14],[15]. Specifically, NIRS has been used to discriminate between and predict the nutrient composition of bamboo plant parts and species, with the goal of developing a method of studying giant panda foraging ecology [16]. Since Foley et al. [17] reviewed applications of NIRS in ecological research, his group has taken the technique a step further and directly predicted forage intake by marsupials from near infrared (NIR) spectra of *Eucalyptus* foliage [18],[19]. Moreover, analysis of stomach contents in dugongs (*Dugong dugon*; [20]) and esophageal extrusa in livestock [21] provided accurate determinations of major dietary components and a logical progression from the direct analysis of forage, to the indirect analysis of feces as a means of determining herbivore dietary characteristics [22],[23],[24],[25].

Fecal near infrared spectroscopy (FNIRS) has also been used to differentiate between species, age, sex, and reproductive status in wild and domestic animals [26]. Here, we hypothesized that FNIRS can be applied to detect biological differences between the feces of male and female giant pandas, between age classes, and among females of different reproductive states. If successful, this will be the first step in developing a powerful non-invasive tool for wildlife population surveys and management that may be applicable to pandas as well as a variety of threatened and endangered species.

## Materials and Methods

Whole fecal samples were collected opportunistically across seasons for 2 years from eight adult (4 male; 4 female) and four juvenile (2 male; 2 female) giant pandas housed in the United States between 2006 and 2007 (Table 1). This included 2 adults at the Memphis Zoo, 2 adults and 1 juvenile at Zoo Atlanta, 2 adults and 2 juveniles at the San Diego Zoo, and 2 adults and 1 juvenile at the Smithsonian's National Zoo. Schaller et al. [4] defines a juvenile as <4 yrs old and in the process of being weaned, while an adult animal is >5 years old and is characterized by increased levels of testosterone for males or a demonstrated prominent estrus cycle for females. One of the adult females was pregnant during a portion of the study and gave birth to a live cub. Bamboo diets differed between institutions (Table 1), likely resulting in a range of fecal chemical composition. For instance, different bamboos fed to captive giant pandas in our study group included *Phyllostachys nuda*, *Pseudososa japonica* (Memphis), *Phyllostachys aurea*, *Bambusa oldhami*, *B. ventricosa* (San Diego), *Arundinaria gigantea*, *P. aureosulcata*, and *P. nigra henon* (Zoo Atlanta), and *P. bissetii* (National Zoo, Washington DC). Additionally, giant pandas seasonally select different bamboo parts consuming mostly culm (and shoots if available) in the spring and leaves throughout the summer, fall and winter [27].

**Table 1:** Bamboo species offered and mean daily consumption for captive giant pandas (*Ailuropoda melanoleuca*) housed in US zoos.

Institution	Giant panda			Mean daily consumption (%)		
Bamboo species fed^a^	ID No.	Age class	Sex	Biscuit	Produce	Bamboo
San Diego Zoo	371	Ad	F^b^	8.3	4.4	87.3
2–6	415	Ad	M	4.7	3.5	91.8
	596	juv	F	3.8	3.3	92.9
	563	juv	M	3.0	2.6	94.3
National Zoo	473	Ad	F	3.1	4.6	92.3
6, 8, 14	458	Ad	M	6.2	5.3	88.5
	595	juv	M	3.7	3.1	93.2
Memphis Zoo	507	Ad	F	2.1	1.6	96.3
5, 6, 8, 9, 12–14	466	Ad	M	1.5	2.2	96.3
Zoo Atlanta	452	Ad	F	3.4	3.5	93.1
1, 6–8, 10, 11, 13, 14	461	Ad	M	4.1	3.5	92.4
	649	juv	F	4.5	3.0	92.4

a Bamboo species fed: 1) *Arundinaria gigantea*, 2) *Bambusa oldhami*, 3) *B. ventricosa*, 4) *B. vulgaris vittata*, 5) *Phyllostachys aurea*, 6) *P. aureosulcata*, 7) *P. bambusoides*, 8) *P. bissetii*, 9) *P. glauca*, 10) *P. nigra* ‘black’, 11) *P. nigra* ‘Henon’, 12) *P. nuda*, 13) *P. rubromarginata*, 14) *Pseudosasa japonica*.

b Adult female pregnant during some period of the study, and which subsequently gave birth.

Zoo staff at the respective institutions collected giant panda fecal samples year-round during normal daily care activities. Samples were placed in clean plastic bags, sealed, and labeled with the panda ID, time, and date. Fecal samples were immediately frozen after collection and sent to the Grazingland Animal Nutrition Lab at Texas A&M University (College Station, TX) in sealed styrofoam coolers. For this initial study, we were not able to control for species of bamboo fed, nor were zoo staff asked to select for specific fecal boli representing different plant part consumption (i.e. bamboo leaf versus culm); instead samples were collected randomly. Thus, our original goal was to determine if physiological discriminant equations could be developed from all samples collected without accounting for diet preferences such as: (1) those that were artificially created by the keepers who selected the bamboo for feeding; (2) samples collected by the keepers with different quantities of plant parts or supplemental food items used during training; and (3) seasonal plant part selection by the bears themselves. However, our subsequent field experience with wild and captive pandas caused us to re-examine our original intent and to also analyze a large (n=105) subset of fecal samples that originated from diets consisting of predominately bamboo leaf.

Fecal samples were processed as previously described by Lyons and Stuth [22]. In brief, whole samples were dried in a forced-air oven at 60°C for 24 hours and subsequently blended and ground in an Udy Mill to pass uniformly through a 1 mm screen for greater homogeneity in particle size [28]. Prior to analysis, ground samples were re-dried at 60°C for at least 3 hours and put in a desiccator for one hour to stabilize sample temperature and moisture. Ground samples were then manually packed in sample cups with a quartz cover glass at a consistent level and compression. Initially, each sample was scanned using a Foss NIRS Systems 6500 Spectrometer (Foss North America, Eden Prairie, MN, USA) with spinning cup attachment. Measurements of reflectance were made over the visible and near infrared ranges (400 to 2500 nm). Samples were later returned to the Memphis Zoo and spectra (350 to 2500 nm) obtained again using an ASD FieldSpec3 portable Spectrophotometer (Analytical Spectral Devices, Boulder, CO, USA) for subsequent analysis.

Spectra were grouped by sex (Experiment 1), age class (Experiment 2), or female reproductive status (Experiment 3) for the appropriate discriminations. Pregnancy was determined by the respective zoo staff using a combination of behavioral observations, hormone level monitoring [29], confirmation by ultrasound [30],[31],[32],[33] and ultimately, birth of a cub. Retrospectively, there were two dietary factors that we hypothesized may impact our ability to predict sex, age, or reproductive status. The first factor was the species of bamboo being offered to the pandas at each institution but we did not achieve a testable distribution of this factor in our initial design. The second, and dominant factor, was the type of plant part consumed (leaf, culm/stalk, or shoot). We thus controlled for plant part in a subsequent analysis by selecting fecal samples derived from a bamboo leaf-dominated (>90%) diet. Such samples are easily determined visually or by spectroscopic analysis in the visible range (∼400 to 100 nm; Vance, unpublished data). These “leaf only” samples were used to generate an additional male∶female discriminant calibration (Experiment 4).

Discrimination between groups was accomplished for Experiments 1 through 3 using WinISI II v. 1.04a software, that utilizes the two-block partial least squares method [28]. This method predicts a set of indicator variables that are assigned to the calibration spectra as follows: samples associated with group A (e.g., male) are labeled {1, 2} in the algebraic matrix and conversely, samples associated with group B (e.g., female) are {2, 1}. “Unknown” samples predicted using the resulting calibration will be assigned to the higher predicted indicator variable of the pair. We used the software default criteria in that a predicted indicator value greater than 1.5 was required for a “correct” determination of sex, age class, or pregnancy status. For example, if the predicted value of sample X is {1.3, 1.7}, the sample is associated with group B. The strength of the group membership increases the closer the value of the respective indicator variable is to 2.0. Experiment 4 calibrations were generated using Grams version 9.1 from Thermo Fisher Scientific.

All discriminant equations were developed using log 1/reflectance (∼400 to 2500 nm) spectra with a mathematical pre-treatment of a second order derivative and scatter correction. Calibrations to classify samples by sex were made using 118 fecal samples collected from adult males and 121 fecal samples from adult females. A similar discrimination was developed using 50 juvenile male samples and 43 samples from juvenile females. In order to weight age classes in the calibration equally, the age class discrimination was created using 100 adult samples randomly selected from the total of 239, versus 93 juvenile panda samples. A limited number of fecal samples were available from the pregnant female (n=8) for comparison with adult females that were not pregnant. To again weight the calibration equivalently, 10 samples from within the adult, not-pregnant female population were randomly selected for this comparison.

Prediction model validation was accomplished in stages. First, “leave one out” cross validation [34] was performed on an entire calibration set. Secondly, we randomly selected and removed 25% of the calibration set, and then used the remaining 75% to create a new discriminant calibration with which to predict the respective validation samples. This calibration using 75% of each respective sample set will be referred to as a “reduced” calibration. Due to low sample size, cross validation on the full set was employed with the female reproductive status calibration and then 10 randomly selected samples from not-pregnant adult females, not used in the original calibration, were used as a validation set. Next, (on the male∶female calibration set only) we performed a “round robin” series in which 3 zoos were used as the calibration set and the fourth zoo used as a validation set. In this exercise the percent of samples making up the validation set ranged from 7% in the case of San Diego Zoo to 67% in the case of Memphis Zoo, rather than an across the board 25%.

A final evaluation was performed in that discrimination was attempted between samples within a broad classification (i.e., sex or age class) that were randomly assigned to an arbitrary group irrespective of any biological significance. This random selection and subsequent group assignment is not to be confused with selection of random samples serving as validation sets for particular calibrations based on “biological” traits; or with similar steps taken to create equally weighted calibrations. The same validation steps (i.e. cross validation and 75% calibration versus 25% validation split) were applied to evaluate the effectiveness of calibrations based on these random “non-biological” groupings. Differences in proportion of fecal spectra that were correctly identified within groups (biological or arbitrary) as compared to a 50% chance of success were determined using Chi-square procedures [35].

## Results

### Experiment 1: Male∶Female discrimination

The original (i.e. all samples, not classified by plant part) adult male∶female discrimination calibration was moderately successful with approximately 80% grouped correctly (P<0.01) for both sexes within the full calibration set (Table 2). Prediction success declined for both males and females after the removal of validation samples. Within the reduced calibration set, samples were 73% (P<0.01) and 65% (P<0.05) correctly predicted for females and males, respectively. Predicting the withheld samples using the reduced calibration equation resulted in 53% of the females (P>0.1) and 72% of the males (P<0.05) predicted correctly. When samples from each unique combination of 3 zoos were used to develop adult male∶female calibrations, success rate averaged 72% for males and 80% for females (P<0.01; Table 3). Validation of these calibrations using the 4^th^ respective zoo in a “round-robin” fashion, however, resulted in correct identifications of only ∼40%.

**Table 2:** Application of near infrared spectroscopy of feces to discriminate between adult male and female captive giant pandas (*Ailuropoda melanoleuca*) housed in US zoos.

	Calibration^b^		Validation^c^	
Discriminant Model^a^	Correct M	Correct F	Correct M	Correct F
M vs. F 100%	78 (92/118)**	81 (98/121)**	NA	NA
M vs. F 75%	65 (58/89)*	73 (66/91)**	72 (21/29)†	53 (16/30)

	Correct A	Correct B	Correct A	Correct B
Random 100%	43 (51/120)	58 (69/119)	NA	NA
Random 75%	49 (44/90)	54 (48/89)	37 (11/30)	60 (18/30)

a Results are reported as: % correct identifications (number correct/group total). Within each discriminant model group, calibrations were developed using either the entire calibration set (100%), or with a reduced set (75%) after removing a randomly selected 25% of samples to be used as a validation set. A, B=random group.

b Refers to the prediction of group membership for each sample in the calibration set itself.

c Refers to the prediction of group membership for each sample in the validation set. NA=not applicable, i.e. there were no validation samples removed from the 100% calibration.

† Within a group, percentage of correct identifications differ from 50% (P<0.1).

* Within a group, percentage of correct identifications differ from 50% (P<0.05).

** Within a group, percentage of correct identifications differ from 50% (P<0.01).

**Table 3:** Effect of zoo on the ability of near infrared spectroscopy of feces to discriminate between adult male and female captive giant pandas (*Ailuropoda melanoleuca*).

	Calibration^b^		Validation^c^	
Discriminant Model^a^	Correct M	Correct F	Correct M	Correct F
Atlanta	78 (78/100)**	79 (78/99)**	28 (5/18)	32 (7/22)
National	74 (78/106)**	82 (89/109)**	25 (3/12)	67 (8/12)
San Diego	75 (82/110)**	81 (91/113)**	38 (3/8)	38 (3/8)
Memphis	63 (24/38)	81 (34/42)**	81 (65/80)**	33 (26/79)

a The discriminant model is labeled after the *validating* zoo, i.e. if the Atlanta Zoo samples are the validating set, then the calibration set is made up of samples from the remaining zoos.

b, c There are a total of 239 samples. The proportion of calibration to validation samples varies with each validation, i.e. Atlanta (83∶17%), National (90∶10%), San Diego (93∶7%) and Memphis (33∶67%).

** Within a group, percentage of correct identifications differ from 50% (P<0.01).

Male versus female was also discriminated within the juvenile age class (Table 4). In the full calibration, successful identifications were 98% for males (P<0.01) and 93% for females (P<0.01). Results from the reduced calibration were 92% and 91% for male and female samples respectively (P<0.01). Validation samples were correctly identified by applying the reduced calibration at 100% (male, (P<0.01) and 82% (female, (P<0.05).

**Table 4:** Application of near infrared spectroscopy of feces to discriminate between juvenile male and female captive giant pandas (*Ailuropoda melanoleuca*) housed in US zoos.

	Calibration^b^		Validation^c^	
Discriminant Model^a^	Correct M	Correct F	Correct M	Correct F
M vs. F 100%	98 (49/50)**	93 (40/43)**	NA	NA
M vs. F 75%	92 (35/38)**	91 (29/32)**	100 (12/12)**	82 (9/11)†

	Correct A	Correct B	Correct A	Correct B
Random 100%	51 (24/47)	50 (23/46)	NA	NA
Random 75%	51 (18/35)	65 (22/34)	42 (5/12)	50 (6/12)

a Results are reported as: % correct identifications (number correct/group total). Within each discriminant model group, calibrations were developed using either the entire calibration set (100%), or with a reduced set (75%) after removing a randomly selected 25% of samples to be used as a validation set. A, B=random group.

b Refers to the prediction of group membership for each sample in the calibration set itself.

c Refers to the prediction of group membership for each sample in the validation set. NA=not applicable, i.e. there were no validation samples removed from the 100% calibration.

† Within a group, percentage of correct identifications differ from 50% (P<0.1).

** Within a group, percentage of correct identifications differ from 50% (P<0.01).

### Experiment 2: Age class discrimination

For age class discrimination, adult samples in the full calibration were predicted 85% correctly (P<0.01) while the juveniles were predicted at 89% (P<0.01), Table 5). Calibration success remained essentially the same for age class discrimination after the validation set removal with 88% of adult samples and 83% of juvenile samples correctly predicted (P<0.01) within this reduced calibration model. Predicting the withheld samples using the new calibration resulted in similar results, as 88% of the adults and 87% of the juvenile samples were classified correctly (P<0.01).

**Table 5:** Application of near infrared spectroscopy of feces to discriminate between adult and juvenile captive giant pandas (*Ailuropoda melanoleuca*) housed in US zoos.

	Calibration^b^		Validation^c^	
Discriminant Model^a^	Correct Ad	Correct juv	Correct Ad	Correct juv
Ad vs. juv 100%	85 (85/100)**	89 (83/93)**	NA	NA
Ad vs. juv 75%	88 (66/75)**	83 (58/70)**	88 (22/25)**	87 (20/23)*

	Correct A	Correct B	Correct A	Correct B
Random 100%	57 (55/97)	35 (34/96)*	NA	NA
Random 75%	56 (41/73)	42 (30/72)	38 (9/24)	50 (12/24)

a Results are reported as: % correct identifications (number correct/group total). Within each discriminant model group, calibrations were developed using either the entire calibration set (100%), or with a reduced set (75%) after removing a randomly selected 25% of samples to be used as a validation set. A, B=random group.

b Refers to the prediction of group membership for each sample in the calibration set itself.

c Refers to the prediction of group membership for each sample in the validation set. NA=not applicable, i.e. there were no validation samples removed from the 100% calibration.

* Within a group, percentage of correct identifications differ from 50% (P<0.05).

** Within a group, percentage of correct identifications differ from 50% (P<0.01).

### Experiment 3: Female reproductive status discrimination

Prediction results for the two reproductive categories within the full calibration set were 75% of pregnant (P>0.1) and 70% of not-pregnant (P>0.1) samples predicted correctly (Table 6). Prediction of the 10 validation samples from not-pregnant females was different (P=0.06) in that 8 were correctly identified as being not-pregnant while 2 were misidentified.

**Table 6:** Application of near infrared spectroscopy of feces to discriminate between pregnant and not pregnant female captive giant pandas (*Ailuropoda melanoleuca*) housed in US zoos.

	Calibration^b^		Validation^c^	
Discriminant Model^a^	Correct Preg	Correct Not	Correct Preg	Correct Not
Pregnant vs. Not	75 (6/8)	70 (7/10)	NA	80 (8/10)†

	Correct A	Correct B	Correct A	Correct B
Random	56 (5/9)	33 (3/9)	NA	NA

a Results are reported as: % correct identifications (number correct/group total). Preg=pregnant, not=not pregnant, A, B=random group.

b Refers to the prediction of group membership for each sample in the calibration set itself.

c Refers to the prediction of group membership for each sample in the validation set. NA=not applicable, i.e. there were no validation samples removed from the calibration, or in the case of pregnant validation samples, none were available.

† Percentage of correct versus incorrect identifications differ (P<0.1).

### Experiment 4: Male∶Female discrimination within “leaf only” calibration set

Using the “leaf only” calibrations, 93% of male and 95% of female samples were correctly identified (P<0.01) within the full calibration set (Table 7). Compared to Experiment 1, prediction success (93%) did not decline (P<0.01) after the removal of validation samples. Predicting the withheld samples using the reduced calibration equation resulted in 87% of the males (P<0.01) and 100% of the females (P<0.01) predicted correctly.

**Table 7:** The effect of using fecal samples derived from “leaf only” bamboo diets on the ability of near infrared spectroscopy to discriminate between adult male and female captive giant pandas (*Ailuropoda melanoleuca*).

	Calibration^b^		Validation^c^	
Discriminant Model^a^	Correct M	Correct F	Correct M	Correct F
M vs. F 100%	93 (62/67)**	95 (36/38)**	NA	NA
M vs. F 75%	93 (41/44)**	93 (27/29)**	87 (20/23)**	100 (9/9)**

	Correct A	Correct B	Correct A	Correct B
Random 100%	49 (26/53)	48 (25/52)	NA	NA
Random 75%	50 (20/40)	64 (25/39)	46 (6/13)	53 (7/13)

a Results are reported as: % correct identifications (number correct/group total). Within each discriminant model group, calibrations were developed using either the entire calibration set (100%), or with a reduced set (75%) after removing a randomly selected 25% of samples to be used as a validation set. A, B=random group.

b Refers to the prediction of group membership for each sample in the calibration set itself.

c Refers to the prediction of group membership for each sample in the validation set. NA=not applicable, i.e. there were no validation samples removed from the 100% calibration.

** Within a group, percentage of correct identifications differ from 50% (P<0.01).

### Random Group Discrimination

Within the adult male and female samples, random calibration, using 120 samples assigned to Group A and 119 samples from Group B, was similar in success to the outcome of a coin toss (approximately 50%; (P>0.1) Table 2). The reduced random calibration samples were 49% and 54% correctly predicted (P>0.1) against the calibration equation for Groups A and B, respectively. Predicting the randomly withheld 25% with the 75% calibration equation resulted in 37% of Group A and 60% of Group B predicted correctly (P>0.1). Similar results were observed for the other “random” calibrations (Tables 3 to 7).

## Discussion

This study indicates that FNIRS could be applied to discriminate between different physiological groups of giant pandas, i.e. sex, age, and female reproductive status. Further validation of this technology should demonstrate its potential as a non-invasive method for wild panda management and population monitoring. However, as indicated by the “round robin” exercise, diet will clearly have an impact on our ability to predict physiological traits in the giant panda and future calibrations will need to take diet, and specifically plant part, into consideration. For example, while strong discriminations were generated for adult male∶ female when only leaf-based samples were used, calibration performance may have improved if bamboo species had been consistent. This scenario is unlikely for pandas in captive facilities; however, in the wild, giant pandas across certain mountain ranges (e.g. Qinling in Shaanxi Province) will all consume the same species of bamboo seasonally, which would facilitate calibration development.

**Figure 1:**
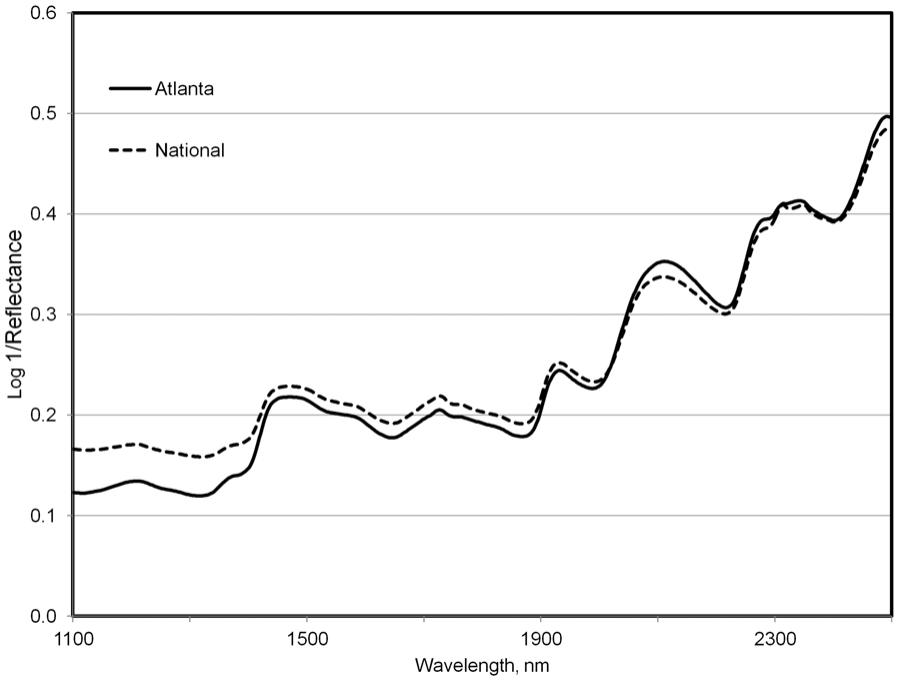
Average fecal near infrared spectra (log 1/reflectance, derivative=0, gap=0) from adult pandas at two different US zoos.

**Figure 2:**
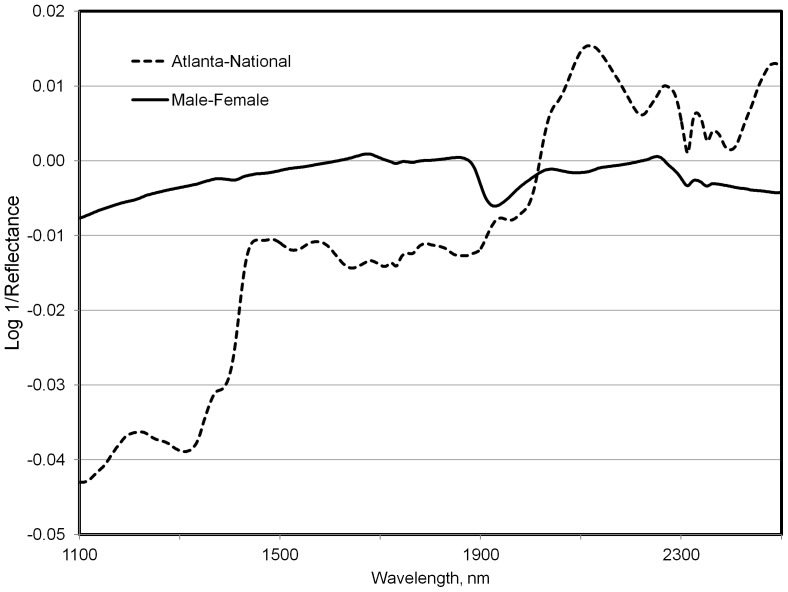
Fecal near infrared difference spectra (log 1/reflectance, derivative=0, gap=0) from adult M-F pandas versus all pandas from two zoos.

Walker et al. [36] found that although spectral differences in domestic goat (*Capra hircus*) feces were affected by age, breed, and sex; the dominant factor was diet. The giant pandas in our study consumed a mix of bamboo leaf, culm, and to a lesser extent diet supplements (i.e. fruit or “biscuits”). Additionally, diets across zoos were from different bamboo species. Table 1 indicates that the animals from the National Zoo consumed a subset of the species consumed by those at Zoo Atlanta. In Figure 1, we present the average fecal spectra from adult pandas at these respective zoos, not accounting for diet selection. A comparison of difference spectra is illustrated in Figure 2. A discriminant equation developed using the samples from the Atlanta (n=47) and National (n=64) zoos correctly predicted 9 of 10 and 8 of 10 validation samples from each zoo respectively (P<0.1). Consistent with the findings of Walker et al. [36] the separation between fecal spectra collected at the two zoos (i.e. diet) is greater than that observed between the two sexes. In our “round-robin” exercise, successful identification of fecal samples ranged from 32 to 67% for females and from 25 to 81% for males. Although diets were from different bamboo species, fecal spectra were reasonably similar as indicated by Mahalanobis distance [37] values. These values for each individual zoo's validation spectra were 2.4±0.2 (Atlanta), 1.9±0.1 (Memphis), 1.6±0.1 (National), and 1.7±0.2 (San Diego). Values for Mahalanobis distance greater than 3.0 are typically used to identify spectra as outliers from the calibration set [38]. Misidentified samples were largely those consisting of mostly bamboo culm rather than leaf, and or animals consuming higher proportions of diet supplements (author's personal observation). Predictions of male versus female were still moderately successful when all zoos/diets were combined in the original calibrations.

Interestingly though, our subsequent experience with pandas in the wild combined with the previous discussion on the effects of diet on fecal NIR spectra, and more specifically the contribution made by plant part (leaf versus culm) leads us to conclude that: 1) bamboo plant part has a greater effect on fecal NIR spectra than bamboo species, 2) fecal samples in the wild can easily be identified visually as consisting of predominately leaf or culm, and 3) that using fecal samples derived from a leaf-dominated diet could result in greater discriminant ability via FNIRS, as compared to that observed with “mixed” samples. We therefore conducted the additional “leaf only” analysis to examine the potential effect of seasonal diet choices afforded pandas in the wild. The greater success rate for these latter discriminations as compared to the “mixed diet” calibrations indicates that application of NIRS as described here may prove to be more applicable in the wild than in captivity. Overall, this initial study on captive giant pandas demonstrated the potential for using FNIRS to distinguish between physiological classes in an ursid species.

Previous studies with captive cervids have shown that fecal spectra from males and females of the same species were significantly different. Our data indicate that we could determine giant panda sex on average 80% of the time for “whole” diets and greater than 90% of the time for leaf-based diets, which is similar to previous reports for pastured red deer (*Cervus elaphus*) and fallow deer (*Dama dama*) [39]. Interestingly, the calibrations for male∶female determination in giant pandas are stronger than those reported for pen-fed white-tailed deer at approximately 70% (Osborn, personal communication). Application of FNIRS to discriminate males from females may not work in all herbivores as the technology has to date proven unsuccessful for the eastern grey kangaroo (*Macropus giganteus*; Foley and Billing, unpublished data). Percent correct determinations for male and female kangaroos were similar to those observed for random groupings in that study. Although male∶female and reproductive status discrimination in livestock has been reported [26], research with cattle [22] found that there was no effect of physiological status of female cattle on FNIRS predictions of diet quality. Taken together, these conflicting results suggest there may be location and species-specific differences in the ability of FNIRS to discriminate between males and females.

**Figure 3:**
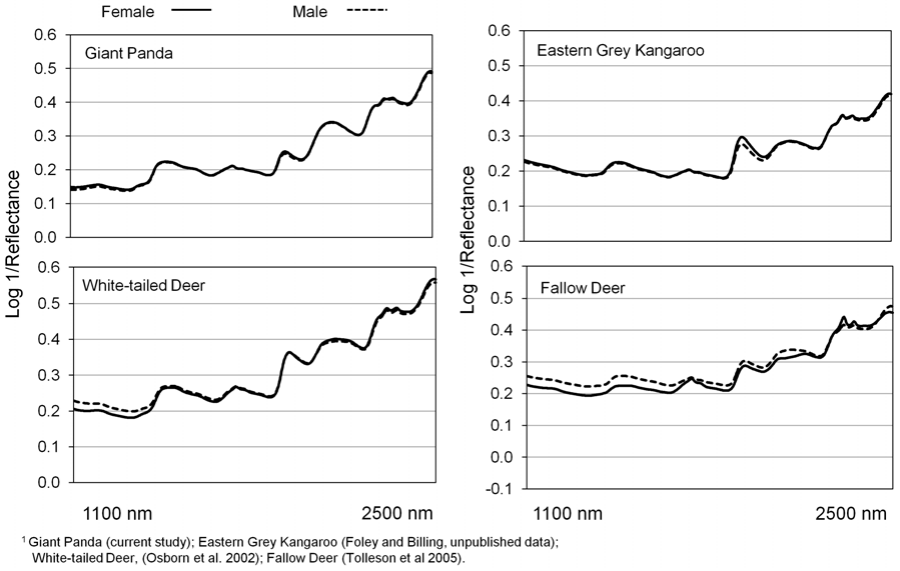
Average fecal near infrared spectra (log 1/reflectance, derivative=0, gap=0) from male and female animals of four different herbivore species.

**Figure 4:**
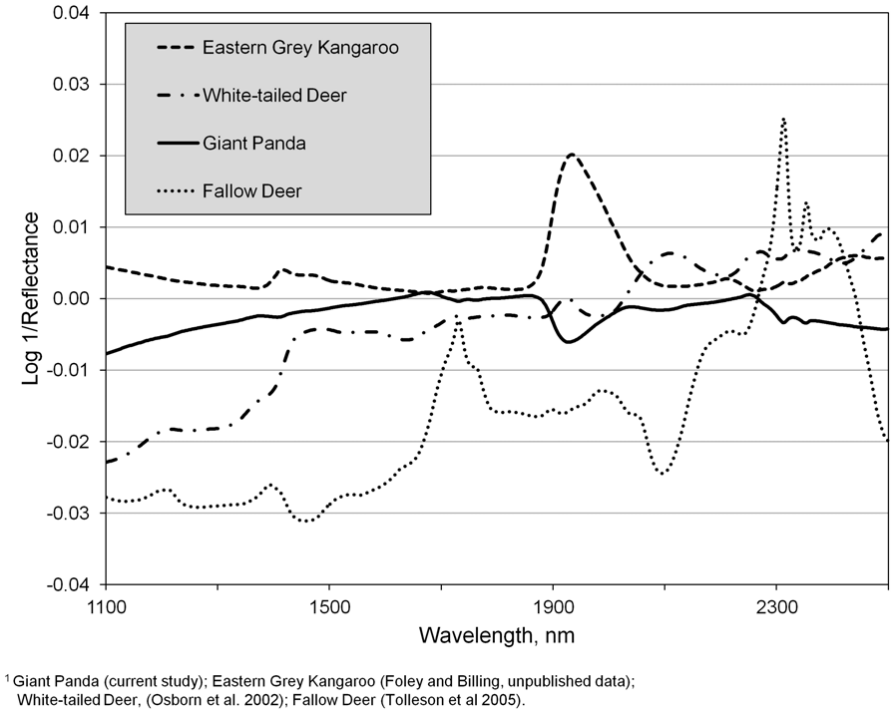
Fecal near infrared difference (male – female) spectra (log 1/reflectance, derivative=0, gap=0) from four different herbivore species.

For illustrative purposes, Figure 3 displays average FNIRS spectra from 3 of these aforementioned studies in comparison with the giant panda. Visual inspection of absorbance spectra for these species does not indicate obvious spectral differences between male and female except for those from fallow deer which also coincides with the greatest discriminant ability reported. When one then examines average difference spectra between male and female at 1100 to 2500 nm for fecal samples from these species; subtle departures become more evident (Figure 4). Again, the difference spectrum from fallow deer exhibits the greatest overall spectral separation and except for the water absorption region around 1900 nm, the kangaroo spectrum exhibits the least separation. Less intuitive is the observation that while the difference between male and female panda spectra appears to be less than that for white-tailed deer, we observed slightly better discriminant ability in the former. Mathematical range and variation, as well as sample number and the number of different individual animals sampled must of course be taken into consideration; therefore, a definitive determination of the ability of FNIRS to discriminate sex in various herbivores will require an experiment designed to incorporate these factors.

The average age class discrimination results were similar to the average values for males versus females on whole diets (not separated by plant part) and indicate that FNIRS can successfully differentiate between juvenile and adult giant pandas. It is unclear why we observed greater discrimination for juvenile animals compared to adult animals in our study. Reasons for the discrepancy in male∶female predictions between adult and juvenile animals may be related to the number of juveniles available for the study compared to adult animals (4 versus 8, respectively) as the sex ratios were identical. As more animals are added to the calibration library for juveniles we will have a better understanding of whether FNIRS can also be used to identify sex in younger animals.

Both adults and juveniles discriminated with a reasonably high prediction rate and this is most likely due to physical rather than chemical differences in the samples, i.e. chewing and processing of bamboo. In their comparison of red and fallow deer, Tolleson et al. [39] proposed that differences in fecal spectra between sexes might be due to such factors as diet selection, retention time, or dentition. An additional explanation of spectral differences between juvenile and adult feces may be hormonal differences resulting from a lack of sexual maturity reached in the juveniles. Pandas do not reach sexual maturity until they are at least 4.5 years old [4]. Hormone levels, in particular steroids, and associated reproductive behaviors displayed by breeding adults that exhibit a normal estrus are different from juvenile pandas [30], [40]. These hormonal differences resulting from sexual maturity, or lack thereof, may be reflected in spectral differences between the fecal samples of various age classes. We should be clear to state that we do not ascribe potential differences in fecal spectra to the ability to measure hormones in minute amounts via FNIRS but rather that the presence of these hormones are likely exerting an effect on ingestive behavior, metabolism, absorption, etc [41], that could be manifest in fecal chemistry and thus, fecal spectra.

The results for our pregnant versus not-pregnant discrimination were similar to our average prediction rates for both sex and age-class. Both pregnant and not-pregnant states were predicted equally well in the full calibration set. Although certainly encouraging, the success of these particular discriminations should be viewed with caution considering our low sample size and number of individuals. Prediction of a validation set drawn from the population of samples collected from not-pregnant females was successful (80%). There are of course a limited number of pregnant pandas in the U.S. each year to obtain samples from. Historically, detection of pregnancy in female pandas has not been possible until the last three weeks prior to birth (using ultrasound) primarily due to female pandas experiencing embryonic diapause in addition to pseudo-pregnancy, even if not bred, which closely resemble the hormone profiles of true pregnancies [31],[32],[33],[40]. Early pregnancy detection would be an extremely valuable tool for captive management as well as monitoring females in the wild. More research is needed to track spectral changes of feces throughout the full reproductive cycle and pregnancy to determine whether we will be able to apply this technique in the field.

When sample libraries were reorganized using random number generators to confirm our categorical spectral separations and calibrations, the percentages for each discrimination were approximately 50%, or the same as a coin toss. This random calibration analyses lends additional strength to our findings that we are in fact identifying specific spectral differences in the physiological parameters we defined for giant panda feces, i.e. the results are due to biology rather than math. Here we demonstrate for the first time that FNIRS is a potential tool for giant panda management. Current calibration equations are robust enough for field testing although steps need to be taken to account for seasonal diet influences and plant part preference by the pandas [27]. An important next step is to acquire fecal samples from wild and captive pandas in China. Research to evaluate the effectiveness of FNIRS to monitor nutrition and physiology of free-ranging ranging pandas is underway. If successful, all three of these discriminations (sex, age, and pregnancy status) will be extremely valuable for field research and assessing wild population demographics. Being able to non-invasively determine the pregnancy status of a female giant panda also has an important implication for captive populations. By having a more accurate idea of the current demographics of wild panda populations, reserve managers can more effectively tailor their conservation plans for habitat management.
